# Acute biotoxicity assessment of heavy metals in sewage sludge based on the glucose consumption of *Escherichia coli*

**DOI:** 10.1098/rsos.181769

**Published:** 2019-01-23

**Authors:** Yazhu Mi, Xiangyun Tao, Xu Zhang, Youbin Si

**Affiliations:** Anhui Province Key Laboratory of FarmLand Ecological Conservation and Pollution Prevention, School of Resources and Environment, Anhui Agricultural University, Hefei 230036, China

**Keywords:** heavy metals, acute biotoxicity, sewage sludge, personal glucose meter, *Escherichia coli*

## Abstract

As a simple and feasible method for acute biotoxicity assessment, personal glucose meter (PGM) can be successfully applied in the early warning of environmental pollutants in sewage. In this paper, the acute biotoxicity of single and joint heavy metals in sewage and real sludge samples was systematically described based on the glucose metabolism of *Escherichia coli* (*E. coli*). Results indicated that the biotoxicity order of five single heavy metals in sewage was Hg^2+^ > As^3+^ > Cu^2+^ > Zn^2+^ > Cd^2+^. The joint heavy metals of Cu^2+^ + Zn^2+^, Cu^2+^ + Cd^2+^, and Cu^2+^ + Hg^2+^ produced synergistic effects, while Cu^2+^ + As^3+^ and Cd^2+^ + Zn^2+^ possessed antagonistic effects for the combined biotoxicity. In spiked sludge, Cd^2+^ and Zn^2+^ owned higher biotoxicity than Cu^2+^ and As^3+^. Notably, the electroplate factory and housing estate sludge respectively showed the highest and lowest inhibition rates as 57.4% and 17.7% under the real sludge biotoxicity assessment. These results demonstrated that PGM was a sensitive and portable method, which could be widely used for acute biotoxicity assessment of heavy metals in sewage sludge.

## 1. Introduction

According to the China Environment Statistical Yearbook (2017), approximately 135.20 million cubic metres per day of urban sewage was produced in China in 2016 [1]. The various applications of sewage sludge administered to agricultural soil can improve the physical, chemical and biological properties of the soil, such as N, P, K and other micronutrients [2,3], and in some cases, can contribute to causing harm to humans and animals after entering food chain [4,5]. One of the main constraints of sewage sludge use on agricultural soil was its high biotoxicity of heavy metals (As, Cu, Cd, Pb, Zn, Hg, etc.) [6]. Concentrations of Zn, Cu, Pb and Cd heavy metals were measured to monitor the instant pollution in the Golden Horn sediment sludge. The total heavy metal concentrations and range of ratios in sludge samples were critical for potential biotoxicity [7]. Otherwise, Cr^3+^ was 6.41 times higher toxicity than the baseline in aquatic ecotoxicity while Cu^2+^ has the major contribution to terrestrial ecotoxicity in the tannery sludge in Bangladesh [8]. Therefore, it is a challenge to rapidly detect biotoxicity of environmental pollutants in sewage sludge.

In the past decades, many researches of biotoxicity assessment have been mainly focused on the sensitivity and pollutant degradation analysis of aquatic organisms and invertebrates [9–12], toxicity assessment of bioluminescent bacteria in water and atmospheric particulate matter [13,14] and electrochemical detection of microbial respiratory inhibition [15–17]. For example, higher concentration of triclosan has produced great oxidative stress to goldfish under the acidic condition [18]. The uninterrupted application of plasma gas can decrease the bioluminescence of *Photobacterium phosphoreum* [14]. The study of Hg^2+^ ion on DC electrical properties has found that HgCl_2_ salt acted as an inhibitor to *Escherichia coli* (*E. coli*) [16]. In addition, those methods were also characterized by high cost and time-consuming. It is necessary to develop a timely and effectively method for acute biotoxicity assessment, which can play an important role in the early warning of environmental pollutants in sewage sludge.

As a successful electrochemical biosensor, personal glucose meter (PGM) has been widely used to monitor the blood glucose of diabetic patients around world [19]. This system was based on the electric flow of glucose produced in the blood samples, which can be measured by PGM. Nowadays, PGM has also been used to detected biological molecules and microorganisms with other instruments or technologies. For example, PGM coupled with aptamer–invertase biosensor can quantify detected quinine in reclaimed wastewater [20]. PGM combination with $UO_{2}^{2+}$-specific DNAzyme was a sensitive and specific method to quantify Pb^2+^ and $UO_{2}^{2+}$ ions [21]. Based on the combined effect of PGM and DNAzyme-capped mesoporous silica nanoparticles (MSNs), this as-prepared sensing platform has been successfully allowed to detect Pb^2+^ at 1.0 ppm level [22]. PGM combination with monoclonal antibody-functionalized magnetic nanoparticle clusters (MNCs) was a simple and sensitive method for the detection of *Salmonella* bacteria in milk [23]. Moreover, PGM can also determine chloramphenicol (CAP) in animal-derived food, and this method has been successfully applied in the on-site assay [24]. PGM also has a certain development prospect for acute biotoxicity assessment of pollutants (such as As^3+^, Ni^2+^, 2,4-dichlorophenol and 4-chlorophenol), and it can be popularly considered due to the low cost and simple operation characteristics [25,26].

Herein, we optimized the experimental parameters and assessed the acute biotoxicity of heavy metal ions (Cu^2+^, Zn^2+^, Cd^2+^, Hg^2+^ and As^3+^) separately as well as joint mixtures by using PGM method. The microorganisms include *E. coli* and *Bacillus subtilis* (*B. subtilis*). The inhibition rate and toxicity unit (TU) values of this study have successfully testified that PGM method can reflect the genuine biotoxicity of real sludge samples and it was a reliable and simple alternative for acute biotoxicity assessment in sewage sludge.

## 2. Material and methods

### 2.1. Materials and reagents

*E. coli* and *B. subtilis* were bought from China General Microbiological Culture Collection Center (CGMCC). The serial numbers were CGMCC 1.1564 and 1.7740 for *E. coli* and *B. subtilis*, respectively. Copper (II) sulphate pentahydrate (CuSO_4_·5H_2_O; 99.0%), zinc sulphate heptahydrate (ZnSO_4_·7H_2_O; 99.5%), cadmium sulphate 8/3hydrate (CdSO_4_·8/3H_2_O; 99.0%), sodium arsenite solution (NaAsO_2_; 90.0%) and mercurous nitrate dihydrate (Hg_2_(NO_3_)_2_·2H_2_O) were bought from Sinopharm Chemical Reagent Co. Ltd (Shanghai, China). PGM was purchased from Changsha Sinocare Biosensing Incorporated Company, China. All the chemicals and reagents were stored at 4°C in a refrigerator.

The sewage containing five heavy metals (Cu^2+^, Zn^2+^, Cd^2+^, As^3+^ and Hg^2+^) was real-time prepared by the industrial wastewater of sewage treatment plant and deionized water. Each kind of heavy metal sewage was prepared in at least five concentrates gradients. The joint heavy metal sewages of Cu^2+^ + Zn^2+^, Cu^2+^ + Cd^2+^, Cu^2+^ + Hg^2+^, Cu^2+^ + As^3+^ and Cd^2+^ + Zn^2+^ were prepared by adding each heavy metals as the same concentration as in their single experiment. All the concentrates gradients of heavy metal sewage were realized by those reagents (CuSO_4_·5H_2_O, ZnSO_4_·7H_2_O, CdSO_4_·8/3H_2_O, NaAsO_2_ and Hg_2_(NO_3_)_2_·2H_2_O) and deionized water and repeated in triplicate.

### 2.2. Microbial cultures

Five vaccination rings of *E. coli* and *B. subtilis* were inoculated in lysogeny broth (LB) medium, respectively. The LB medium was put into oscillation incubator (180 r.p.m.) and shaken at 30°C for 24 h to obtain 100 ml bacterial saline suspension solution. Bacterial saline suspension solution (10 ml) was taken for subculture three times with the some method as above. The last subculture of *E. coli* and *B. subtilis* was incubated to the post stabilization period. Then 20 ml 0.85% (w/v) saline was added into the bacterial saline suspension solution and centrifuged at 6000 r.p.m. for 5 min. The *E. coli* and *B. subtilis* suspension solution was diluted by saline (0.85(w/v)). The UV spectrophotometer (TU1901, Shanghai) has been used to determine the optical density of bacterial suspension solution at 600 nm (OD_600_) for 2.3. The bacterial suspension solutions were stored at 4°C in a refrigerator for less than 3 h until the experiment started [27,28].

### 2.3. Acute biotoxicity assessment in sewage

The sewage samples were prepared by evenly mixing 1 ml LB medium, 0.1 ml glucose, 0.1 ml heavy metal liquid and 0.8 ml *E. coli* suspension solution. Saline (0.85% (w/v)) was selected as the control sample. All the samples were cultivated at 30°C after a period of time, then centrifuged at 9000 r.p.m. for 1 min. Supernatants (5 µl) were taken out from the suspension solution and the glucose concentration measured by PGM. Based on the initial and final glucose concentration, the equivalent inhibition rate was calculated by equation (2.1) [29].

2.1 $$\mathrm{Inhibition}(\text{\%})=\frac{\left(C_{e}-C_{c}\right)}{\left(C_{i}-C_{c}\right)}\times 100\text{\%},$$

where, *C*_i_ is the initial glucose concentration of all samples; *C*_e_ is the final glucose concentration of control group samples; *C*_c_ is the final glucose concentration when heavy metal existed.

### 2.4. Joint biotoxicity assessment in sewage

The joint biotoxicity assessment was determined by the TU, which has been widely used to test the reaction of chemical mixture. TU values were calculated by equation (2.2) [25].

2.2 $$\sum TU_{i}=\overset{n}{\underset{i=1}{\sum }}\frac{C_{i}}{IC_{50,i}},$$

where, *C_i_* is the concentration of mixture component; *n* is the type of toxic substance in the sample; IC_50,i_ is the half maximal inhibitory concentration of component *i*. Among them, half maximal inhibitory concentration of mixture (IC_50mix_) < 1TU means synergistic effect; IC_50mix_ = 1TU means additive effect; IC_50mix_ > 1TU means antagonistic effect.

### 2.5. Biotoxicity assessment of heavy metals in sludge

Due to the high content and strong biotoxicity of heavy metals in the sewage sludge, the toxicants have exceeded the detection range by microbial method. To improve the sensitivity of the PGM method, spiked sludge was prepared by industrial park sludge and fresh soil with different mixed ratio. Then, added the reagents (CuSO_4_•5H_2_O, ZnSO_4_•7H_2_O, CdSO_4_•8/3H_2_O and NaAsO_2_) were added into the heavy metal extract solution to obtain different concentrates of spiked sludge, respectively.

The heavy metal composition analysis of four real sludge samples (industrial park, electroplate factory, steelworks and housing estate) was performed by inductively coupled plasma-atomic emission spectrometry (ICP-AES) (ICP-5000, Zhejiang). Real sludge samples were directly prepared by four real sludge and fresh soil using the same process as above. All spiked or real sludge samples were air-dried and milled by 2 mm screen.

Samples of air-dried sludge (2 g) and 0.1 mol l^−1^ hydrochloric acid were taken into centrifuge tube, then centrifuged (180 r.p.m.) for 2 h to obtain the heavy metal extract solution. Using the same process as the acute biotoxicity assessment in sewage, biotoxicity assessment of heavy metals in sludge samples were detected by PGM and calculated by equations (2.1) and (2.2). The parameter optimization process of the heavy metal extract solution is shown in electronic supplementary material, figure S1.

### 2.6. Statistical analysis

The statistics box figure and line chart was analysed by Origin 9.0. Data were presented as mean ± standard error of mean (s.e.m.). SPSS 19.0 was used for statistics analysis and the significant difference was at 0.05 probability level.

## 3. Results

### 3.1. Principle verification

**Figure 1.**
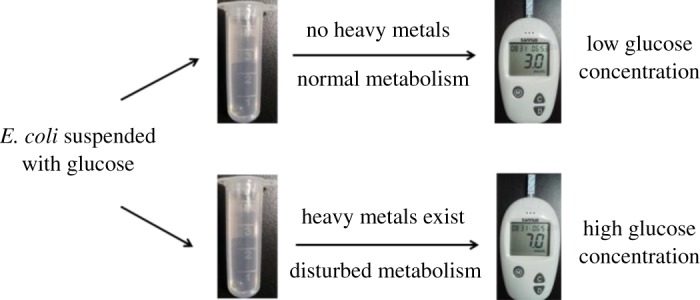
A schematic illustration of experimental procedure by using a PGM for acute biotoxicity assessment based on glucose consumption of *E. coli*.

Glucose oxidase or glucose dehydrogenase of bacteria could be inhibited by heavy metal, which was related with glucose production. As shown in figure 1, when *E. coli* was suspended with glucose and heavy metal was not added, lots of glucose was consumed in normal metabolism and PGM showed the low glucose concentration as 3.0 mM. If heavy metal existed in the suspended solution, glucose will be less consumed in disturbed metabolism. High glucose concentration was displayed in PGM as 7.0 mM.

**Figure 2.**
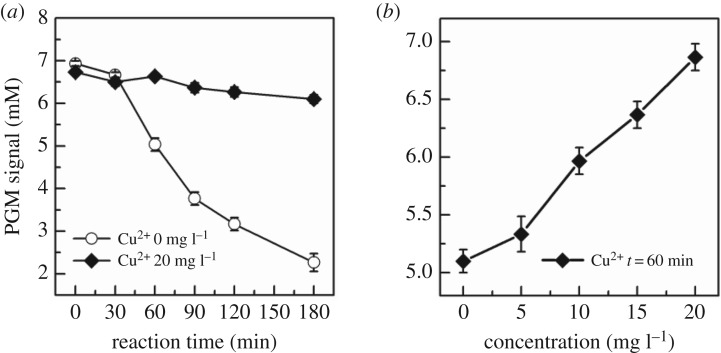
Principle analysis of biotoxicity assessment based on the microbial glucose metabolism: (*a*) the glucose concentration–time curve with or without the existence of Cu^2+^ (20 mg l^−1^); (*b*) the final glucose concentration–Cu^2+^ concentration curve when samples were incubated for 60 min.

In order to verify the glucose concentration, PGM can be applied in acute biotoxicity assessment as an indicator; the PGM signal of *E. coli* and glucose suspended with or without Cu^2+^ is shown in figure 2. Figure 2*a* is the glucose concentration–time curve of the microbial glucose metabolism. PGM signals were 5.03 ± 0.15 mM with no Cu^2+^ and 6.63 ± 0.06 mM with 20 mg l^−1^ Cu^2+^ at 60 min, which has the significant difference between each other. In the non-toxic environment, *E. coli* can consume more glucose than in a toxic environment. In addition, the final glucose concentration–Cu^2+^ concentration curve was observed when samples were incubated for 60 min, as shown in figure 2*b*.

### 3.2. Optimization of experimental conditions

**Figure 3.**
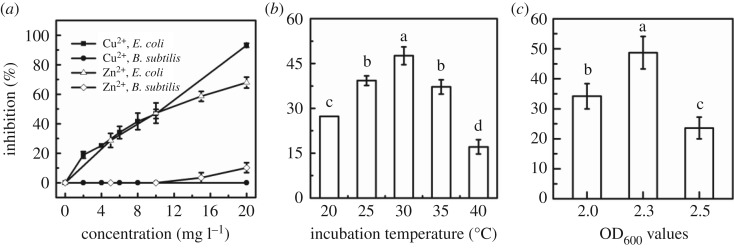
The response of microbe to heavy metals under different experimental conditions: (*a*) *E. coli* and *B. subtilis* to Cu^2+^ and Zn^2+^; (*b*) the incubation temperature of *E. coli* with 10 mg l^−1^ Cu^2+^; (*c*) the OD_600_ values of *E. coli* with 10 mg l^−1^ Cu^2+^. Data points represent the average of three replicates.

Parameter optimization of bioassay in terms of *E. coli* and *B. subtilis* concentration, culture temperature and OD_600_ values for inhibition of glucose metabolism was studied to obtain a better condition for biotoxicity assessment of Cu^2+^ and Zn^2+^. In figure 3*a*, from the responses of *E. coli* and *B. subtilis* to two heavy metal ions, Cu^2+^ and Zn^2+^ had a relatively high inhibitory effect on the glucose metabolism of *E. coli*, while the glucose metabolism of *B. subtilis* was almost not affected. It was observed that with incubation temperature and microbial concentration increased, the inhibition of glucose metabolism on *E. coli* by Cu^2+^ (10 mg l^−1^) increased firstly and then decreased. The highest inhibition was obtained at 30°C and the inhibition rate was 47.6% in figure 3*b*. And figure 3*c* shows that the largest inhibition rate of *E. coli* was 57.4% when microbial concentration (OD_600_ value) was 2.3.

### 3.3. Acute biotoxicity assessment of single heavy metal in sewage

**Figure 4.**
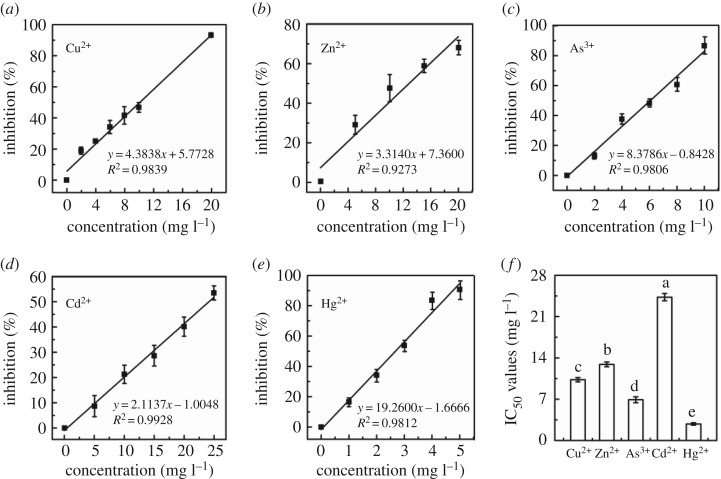
Inhibitory curves of heavy metal (*a*) Cu^2+^, (*b*) Zn^2+^, (*c*) As^3+^, (*d*) Cd^2+^, (*e*) Hg^2+^ at different concentrations under the optimized conditions. Data points represent the average of three replicates. (*f*) IC_50_ values of five single heavy metals obtained from the inhibitory curves.

Biotoxicity of five single heavy metals was assessed after optimization of experimental parameters. As shown in figure 4, with the concentration of heavy metal increased, the inhibition of glucose metabolism on *E. coli* increased. The inhibitory curves of five single heavy metals were adapted to linear fitting. All the R square of linear fitting correlation was greater than 0.9200. The IC_50_ values of Cu^2+^, Zn^2+^, As^3+^, Cd^2+^ and Hg^2+^ were 10.3, 12.9, 6.9, 24.3 and 2.8 mg l^−1^, respectively. The biotoxicity order of single heavy metal to *E. coli* was Hg^2+^ > As^3+^ > Cu^2+^ > Zn^2+^ > Cd^2+^.

Comparing the IC_50_ values of heavy metal in this paper with other methods, many IC_50_ values of Cd^2+^, Zn^2+^, Cu^2+^ and Hg^2+^ to *E. coli* by using glucose consumption inhibition method were lower than other methods [29–32], as shown in table 1. From the comparison of single heavy metal toxicity assessment, it can be seen that Hg^2+^ had the highest biological toxicity for different microbial species with different methods.

**Table 1.** Comparison of IC_50_ values by measuring glucose consumption with other methods obtained in references.

	heavy metals, IC_50_ (mg l^−1^)					
methods	Cu^2+^	Zn^2+^	As^3+^	Cd^2+^	Hg^2+^	references
glucose consumption inhibition, *E. coli*	10.3	12.9	6.9	24.3	2.8	present study
glucose consumption inhibition, *E. coli*	—	—	5.0	14.2	—	[25]
amperometry mixed microbes	16.5	—	—	20.5	—	[29]
colorimetric bioassay, *B. subtilis*	5.0	9.3	—	21.3	—	[30]
amperometry, *E. coli*	44.0	—	—	79.0	21.2	[33]
amperometry, *E. coli*	20.2	53.2	—	36.2	—	[34]
amperometry, *Psychrobacter* sp.	2.6	10.9	—	47.3	0.8	[31]
amperometry, *S. cerevisiae*	10.12	—	—	13.88	—	[35]
nitrification inhibition	41.5	22.6	—	79.0	—	[32]
*Photobacterium phosphoreum*	1.905^a^	—	—	0.537^a^	—	[36]
*Photobacterium phosphoreum*	—	0.5^a^	—	—	—	[37]

^a^15 min IC_50_ (mg l^−1^).

### 3.4. Joint biotoxicity assessment of binary heavy metals in sewage

For real wastewater or other contaminants, chemical substances often existed as mixtures. It was crucial to study the mixture poisons biotoxicity. As shown in table 2, the IC_50_ value of each heavy metal was treated as 1 TU in the mixture for subsequent joint toxicity evaluation. In order to facilitate the setting of binary heavy metal concentration, the concentrations of Cu^2+^, Zn^2+^, Cd^2+^, Hg^2+^ and As^3+^ were defined as A at 10, 20, 20, 10 and 3 mg l^−1^, respectively.

**Table 2.** The TU values of single heavy metal in different concentration gradients. Note: the concentrations of Cu^2+^, Zn^2+^, Cd^2+^, Hg^2+^ and As^3+^ were defined as A at 10, 20, 20, 10 and 3 mg l^−1^, respectively.

concentration (mg l^−1^)	$TU_{Cu^{2+}}$	$TU_{Zn^{2+}}$	$TU_{Cd^{2+}}$	$TU_{As^{3+}}$	$TU_{Hg^{2+}}$
0	0	0	0	0	0
0.2A	0.19	0.31	0.17	0.29	0.21
0.4A	0.39	0.63	0.33	0.58	0.43
0.6A	0.58	0.94	0.50	0.87	0.64
0.8A	0.78	1.25	0.66	1.16	0.86
A	0.97	1.56	0.82	1.45	1.07

For the combined effect experiment, the combined toxicity was measured through the TU of binary compounds in table 3. Based on the response relation of the obtained dose (TU-based), combined effect of heavy metals was defined as synergy effect (IC_50mix_ < 1 TU), additive effect (IC_50mix_ = 1 TU) or antagonism effect (IC_50mix_ > 1 TU).

**Table 3.** The sum of TU of binary heavy metals. Note: A has the same means as in table 2.

concentration (mg l^−1^)	$TU_{Cu^{2+}+Zn^{2+}}$	$TU_{Cu^{2+}+As^{3+}}$	$TU_{Cu^{2+}+Hg^{2+}}$	$TU_{Cd^{2+}+Zn^{2+}}$	$TU_{Cu^{2+}+Cd^{2+}}$
0	0	0	0	0	0
0.2A	0.50	0.48	0.40	0.48	0.36
0.4A	0.99	0.97	0.82	0.96	0.72
0.6A	1.52	1.45	1.22	1.44	1.08
0.8A	2.03	1.94	1.64	1.91	1.44
A	2.53	2.42	2.04	2.38	1.79

**Figure 5.**
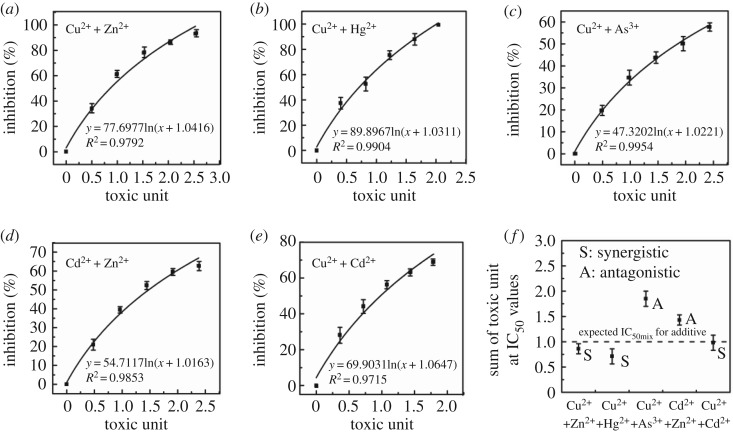
The dose (TU-based) response relationship curves of the binary heavy metals at different concentrations: (*a*) Cu^2+^ + Zn^2+^, (*b*) Cu^2+^ + Hg^2+^, (*c*) Cu^2+^ + As^3+^, (*d*) Cd^2+^ + Zn^2+^ and (*e*) Cu^2+^ + Cd^2+^ under the optimized conditions. Data points represent the average of three replicates. (*f*) The half maximal inhibitory concentration of mixture (IC_50mix_) to microbial cells exposed to binary heavy metals. IC_50mix_ is estimated as toxic unit (unitless). S: synergistic, greater than additivity. A: antagonistic, less than additivity.

In this study, five binary heavy metals mainly produced as synergistic and antagonistic effects in figure 5. The dose (TU-based) response relationship curves of the binary heavy metals at different concentrations were adapted to logarithmic function fitting. All R square of logarithmic function fitting correlation was greater than 0.9750. Synergistic response to *E. coli* occurred when Cu^2+^ + Zn^2+^, Cu^2+^ + Cd^2+^ and Cu^2+^ + Hg^2+^ mixed together because the IC_50mix_ values were detected to be 0.86, 0.98 and 0.71, respectively. Joint heavy metals of Cu^2+^ + As^3+^ and Cd^2+^ + Zn^2+^ produced antagonistic response for the biotoxicity as the IC_50mix_ values were 1.85 and 1.43, respectively.

### 3.5. Acute biotoxicity assessment of heavy metals in sludge

To assess the acute biotoxicity of sewage sludge, the types and concentrations of heavy metals in the sludge were spiked according to the concentration of main heavy metals in industrial park sludge in our designed study. The mixed ratio of real sludge and fresh soil was 1 : 9. Different from sewage, it could produce acute biotoxicity to the bacteria when heavy metal dissolved from the sludge. The higher *E. coli* inhibition in spiked sludge was done by hydrochloric acid than acetic acid and deionized water, as shown in electronic supplementary material, figure S1(a).

**Figure 6.**
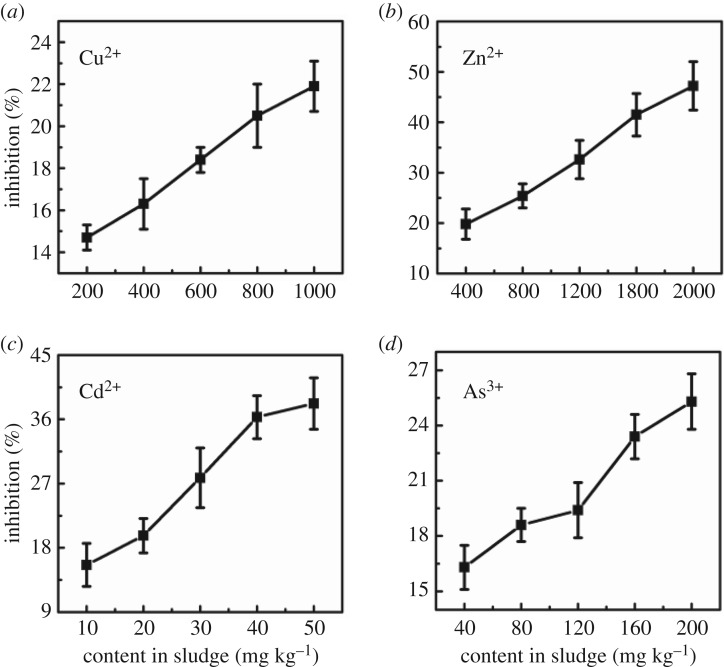
Inhibition response of *E. coli* to heavy metals in sludge at different contents (*a*) Cu^2+^, (*b*) Zn^2+^, (*c*) Cd^2+^ and (*d*) As^3+^ under the optimized conditions. Data points represent the average of three replicates.

As shown in figure 6, the biotoxicity of spiked sludge of Cu^2+^ and As^3+^ was relatively weak to *E. coli*. When the contents of Cu^2+^ and As^3+^ were respectively 1000 and 200 mg kg^−1^, the growth inhibition rate was lower than 25%. However, when the contents of Cd^2+^ and Zn^2+^ were respectively 50 and 2000 mg kg^−1^, the growth inhibition rates were 38.2% and 47.2% to *E. coli*.

### 3.6. Acute biotoxicity assessment of real sludge samples

**Figure 7.**
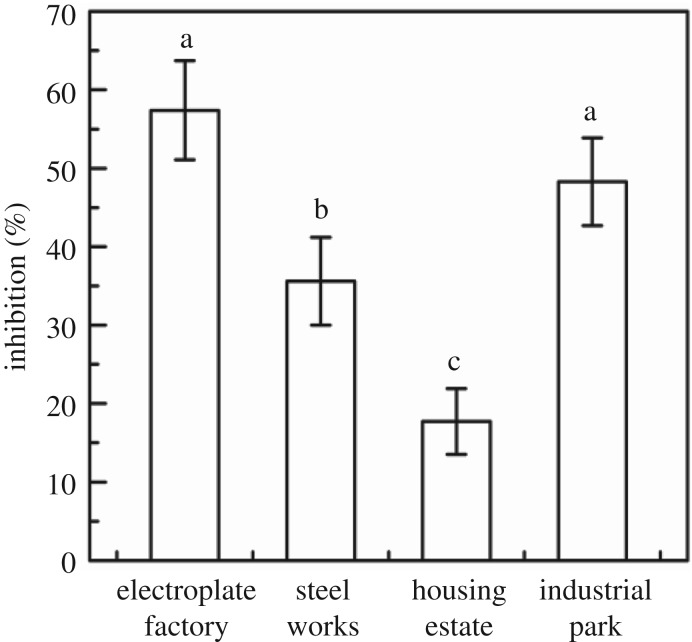
Inhibition rate of *E. coli* to heavy metals in real sludge samples from different sites.

The PGM method was applied in real sludge samples, including industrial park, electroplate factory, steelworks and housing estate. In figure 7, for the sludge from electroplate factory, steelworks, housing estate and industrial park, the inhibition rates on the growth of *E. coli* were 57.4%, 35.6%, 17.7% and 48.3%, respectively. The biotoxicity order of sludge was: electroplate factory > industrial park > steelworks > housing estate. Comparing the inhibition rates on the growth of *E. coli* in real sludge samples with ICP-AES method, electroplate factory and industrial park sludge also have more seriously heavy metal pollution, as shown in table 4.

**Table 4.** The pH values and heavy metal contents of real sludge by ICP-AES method. Note: — undetected.

		heavy metal contents (mg kg^−1^)				
real sludge	pH	Cu	Zn	Cd	As	Hg
electroplate factory	7.32	1283.2	1526.3	2.3	38.9	—
steelworks	5.07	604.1	790.2	1.4	14.8	—
housing estate	6.48	212.4	304.1	—	2.5	—
industrial park	6.23	427.8	5233.8	2.41	147.4	—

## 4. Discussion

In recent years, some researches have indicated that heavy metals and other pollutants inhibited the glucose oxidase or glucose dehydrogenase of bacteria, which were related with glucose production [30,38,39]. A higher PGM signal was detected with the increase of Cu^2+^ and Zn^2+^ concentration [40]. Under the consistent parameter optimization of bioassay in terms of *E. coli* and *B. subtilis* concentration, culture temperature and OD_600_ values [41,42], *E. coli* were more sensitive and suitable for heavy metal biotoxicity assessment in the following studies.

Comparing the IC_50_ values of single heavy metal with the integrated biosensor, which was prepared by benzoquinone (BQ) redox mediator and gelatin-hybrid hydrogel (GSH), the detected IC_50_ values of Cu^2+^, Cd^2+^ and Hg^2+^ to *E. coli* were respectively 44.0, 79.0 and 21.2 mg l^−1^ [33]. Combined with *p*-benzoquinone-mediated amperometric biosensor, the IC_50_ values of Cu^2+^, Zn^2+^, Cd^2+^ and Hg^2+^ to *Psychrobacter* sp. were 2.6, 10.9, 47.3 and 0.8 mg l^−1^, respectively [31]. It was noted that glucose consumption method was more sensitive to heavy metals than various other methods. At the same time, Hg^2+^ had the highest biological toxicity for microbial in different methods [43]. Catterall *et al*. have studied the toxic effect of Hg^2+^ on *E. coli* through respiratory method, and showed that the IC_50_ value was 2.03 mg l^−1^, which was basically consistent with our study [44]. Otherwise, the research work of industrial wastewater under the *Photobacterium phosphoreum* method had shown that Cd^2+^ had a higher biotoxicity than Cu^2+^ [36]. The main reason for the result different from our findings might be that different microorganisms and test procedure had different sensitivity to pollutants.

According to the binary heavy metal biotoxicity research, the combined effects of Cd^2+^ + Zn^2+^ and Cu^2+^ + Cd^2+^ have consistence conclusions in different methods [45,46]. In our studies, combined toxicity of Cu^2+^ + Cd^2+^ also produced synergistic effect, while Cd^2+^ + Zn^2+^ showed as antagonistic effect. This might be ascribed to Cu^2+^ being more bioavailable than other ions, which could enlarged the cell membrane permeability when Cu^2+^ and Cd^2+^ coexist in binary mixture. On the other hand, Zn^2+^ and Cd^2+^ coexistence would decrease the system toxicity due to the formation of less bioavailable complex than both single heavy metals [27].

Finally, we have applied the glucose consumption inhibition method to measure the biotoxicity of heavy metal to *E. coli* in spiked sludge and real samples. Biotoxicity of spiked sludge of Cu^2+^ and As^3+^ was weaker to *E. coli* than Zn^2+^ and Cd^2+^. This might be related to the low proportion of effective state and low biological toxicity of Cu^2+^ and As^3+^ found in sludge by many existing studies. Moreover, the form of Cu was mainly associated with the organic matter and Zn showed the higher proportion of exchangeable and reducible fractions in sludge, which had higher biotoxicity to *E. coli* [8,47]. For the biotoxicity of heavy metal in real samples, colorimetric method, electrochemical biosensor and other methods were applied to the acute biotoxicity detection of real heavy metals, and showed that electroplate factory and industrial park sludge had the higher biotoxicity to bacterial, which was basically consistent with the research results in our work [25,30,35].

On the whole results, it was successfully testified that heavy metal pollution had higher inhibition on the microbial glucose metabolism in this study. Based on the glucose consumption by *E. coli*, acute biotoxicity assessment can be consistent and accurate reflected by using PGM.

## 5. Conclusion

The systems of PGM and *E. coli* can detected the single and joint biotoxicity of heavy metal ions in sewage sludge. The IC_50_ values of Cu^2+^, Zn^2+^, Cd^2+^, Hg^2+^ and As^3+^ were 10.3, 12.9, 24.3, 2.8 and 6.9 mg l^−1^ in sewage, respectively, revealing that Hg^2+^ was the most toxic ion to *E. coli*. The biotoxicity of binary heavy metals and four real sludge samples were also successfully assessed. In conclusion, our study offered an economic, timely and sensitive alternative for acute biotoxicity assessment of pollutants monitoring in sewage sludge.

## Supplementary Material

The parameter optimization of heavy metal extract solution in spiked sludge based on the glucose consumption of Escherichia coli

## Data accessibility

The figures of the study are based on the Origin 9.0. Relevant data and figures are included in the paper or in the electronic supplementary material.

## References

[1] China Environment Statistical Yearbook. 2017 http://www.doc88.com/p-7059188414250.html.

[2] Saha S, Saha BN, Pati S, Pal B, Hazra GC 2018 Agricultural use of sewage sludge in India: benefits and potential risk of heavy metals contamination and possible remediation options—a review. Int. J. Environ. Technol. Manag. 20, 183–199. (10.1504/IJETM.2017.089645)

[3] Koutroubas SD, Antoniadis V, Fotiadis S, Damalas CA 2014 Growth, grain yield and nitrogen use efficiency of Mediterranean wheat in soils amended with municipal sewage sludge. Nutr. Cycl. Agroecosyst. 100, 227–243. (10.1007/s10705-014-9641-x)

[4] Pathak A, Dastidar MG, Sreekrishnan TR 2009 Bioleaching of heavy metals from sewage sludge: a review. J. Environ. Manage. 90, 2343–2353. (10.1016/j.jenvman.2008.11.005) 19303195

[5] Tytła M, Widziewicz K, Zielewicz E 2016 Heavy metals and its chemical speciation in sewage sludge at different stages of processing. Environ. Technol. 37, 899–908. (10.1080/09593330.2015.1090482) 26419833

[6] Rossi G, Beni C 2018 Effects of medium-term amendment with diversely processed sewage sludge on soil humification-mineralization processes and on Cu, Pb, Ni, and Zn bioavailability. Plants 7, 16–25. (10.3390/plants7010016) PMC5874605 29498633

[7] Kanat G, Ikizoglu B, Erguven GO, Akgun B 2018 Determination of pollution and heavy metal fractions in Golden Horn sediment sludge (Istanbul, Turkey). Pol. J. Environ. Stud. 27, 1–7. (10.15244/pjoes/80805)

[8] Juel MAI, Chowdhury ZUM, Ahmed T 2016 Heavy metal speciation and toxicity characteristics of tannery sludge. AIP. Conf. Proc. 1754, 060009-1-6 (10.1063/1.4958450)

[9] Ye N, Wang Z, Wang S, Peijnenburg WJGM 2018 Toxicity of mixtures of zinc oxide and graphene oxide nanoparticles to aquatic organisms of different trophic level: particles outperform dissolved ions. Nanotoxicology 12, 1–16. (10.1080/17435390.2018.1458342) 29658385

[10] Ortizsantaliestra ME, Maia JP, Egeaserrano A, Lopes I 2018 Validity of fish, birds and mammals as surrogates for amphibians and reptiles in pesticide toxicity assessment. Ecotoxicology 27, 819–833. (10.1007/s10646-018-1911-y) 29492806

[11] Huang ZZ et al. 2017 Toxicity mechanisms and synergies of silver nanoparticles in 2,4-dichlorophenol degradation by *Phanerochaete chrysosporium*. J. Hazard. Mater. 321, 37–46. (10.1016/j.jhazmat.2016.08.075) 27607931

[12] Huang ZZ, Chen GQ, Zeng GM, Chen AW, Zuo YN, Guo Z, Tan Q, Song ZX, Niu QY 2015 Polyvinyl alcohol-immobilized *Phanerochaete chrysosporium* and its application in the bioremediation of composite-polluted wastewater. J. Hazard. Mater. 289, 174–183. (10.1016/j.jhazmat.2015.02.043) 25725339

[13] Suwal S, Coronel-Aguilera CP, Auer J, Applegate B, Garner AL, Huang JY 2018 Mechanism characterization of bacterial inactivation of atmospheric air plasma gas and activated water using bioluminescence technology. Innov. Food Sci. Emerg. Technol. S1466856417308895. (10.1016/j.ifset.2018.01.007)

[14] Bodini SF, Malizia M, Tortelli A, Sanfilippo L, Zhou X, Arosio R 2018 Evaluation of a novel automated water analyzer for continuous monitoring of toxicity and chemical parameters in municipal water supply. Ecotoxicol. Environ. Saf. 157, 335–342. (10.1016/j.ecoenv.2018.03.057) 29627418

[15] Yong DM, Liu CY, Zhu CZ, Yu DB, Liu L, Zhai JF, Guo SJ 2015 Detecting total toxicity in water using a mediated biosensor system with flow injection. Chemosphere 139, 109–116. (10.1016/j.chemosphere.2015.05.031) 26071865

[16] Abu-Ali H, Nabok A, Smith T, Al-Shanawa M 2016 Development of electrochemical inhibition biosensor based on bacteria for detection of environmental pollutants. Sens. Biosensing Res. 13, 109–114. (10.1016/j.sbsr.2016.10.007)

[17] Karczmarczyk A, Baeumner AJ, Feller KH 2017 Rapid and sensitive inhibition-based assay for the electrochemical detection of *Ochratoxin A* and *Aflatoxin M1* in red wine and milk. Electrochim. Acta 243, 82–89. (10.1016/j.electacta.2017.05.046)

[18] Li CG, Qu RJ, Chen J, Zhang S, Allam A, Ajarem J, Wang ZY 2018 The pH-dependent toxicity of triclosan to five aquatic organisms (*Daphnia magna*, *Photobacterium phosphoreum*, *Danio rerio*, *Limnodrilus hoffmeisteri*, and *Carassius auratus*). Environ. Sci. Pollut. Res. 25, 9636–9646. (10.1007/s11356-018-1284-z) 29363032

[19] Musacchio N, Ciullo I, Scardapane M, Glancaterinl A, Pessina L, Maino S, Galofatto R, Nicoluccr A, Rossi MC 2018 Efficacy of self-monitoring blood glucose as a key component of a chronic care model versus usual care in type 2 diabetes patients treated with oral agents: results of a randomized trial. Acta Diabetol. 55, 295–299. (10.1007/s00592-017-1072-0) 29138925

[20] Qiu Y, Gu C, Li B, Shi H 2018 Aptameric detection of quinine in reclaimed wastewater by using a personal glucose meter. Anal. Methods 24, 90–98. (10.1039/C8AY00585K)

[21] Xiang Y, Lu Y 2013 An invasive DNA approach toward a general method for portable quantification of metal ions using a personal glucose meter. Chem. Commun. 49, 585–587. (10.1039/c2cc37156a) PMC3765066 23208450

[22] Fu LB, Zhuang JY, Lai WQ, Que XH, Lu MH, Tang DP 2013 Portable and quantitative monitoring of heavy metal ions using DNAzyme-capped mesoporous silica nanoparticles with a glucometer readout. J. Mater. Chem. B 1, 6123–6128. (10.1039/C3TB21155J) 32260997

[23] Joo J, Kwon D, Shin HH, Park KH, Cha HJ, Jeon S 2013 A facile and sensitive method for detecting pathogenic bacteria using personal glucose meters. Sens. Actuators B Chem. 188, 1250–1254. (10.1016/j.snb.2013.08.027)

[24] Chen S, Gan N, Zhang HR, Hu FT, Li TH, Cui H, Cao YT, Jiang QL 2015 A portable and antibody-free sandwich assay for determination of chloramphenicol in food based on a personal glucose meter. Anal. Bioanal. Chem. 407, 2499–2507. (10.1007/s00216-015-8478-8) 25644521

[25] Fang DY, Gao GY, Yu Y, Shen J, Zhi JF 2016 Adaptive use of a personal glucose meter (PGM) for acute biotoxicity assessment based on the glucose consumption of microbes. Analyst 141, 3004–3011. (10.1039/C5AN02478A) 27055358

[26] Ravi C, Naga SKG, Selvaraj N, Sushanta KM 2014 Detection of *Escherichia coli* in potable water using personal glucose meters. Anal. Methods 6, 6223–6227. (10.1039/C4AY01249F)

[27] Gunda NS, Chavali R, Mitra SK 2016 A hydrogel based rapid test method for detection of *Escherichia coli* (*E. coli*) in contaminated water samples. Analyst 141, 2920–2929. (10.1039/C6AN00400H) 27137782

[28] Westphal LL, Lau J, Negro Z, Moreno IJ, Mohammed WI, Lee H, Tang HX, Finkel SE, Kram KE 2018 Adaptation of *Escherichia coli* to long-term batch culture in various rich media. Res. Microbiol. 169, 145–156. (10.1016/j.resmic.2018.01.003) 29454026

[29] Gao GY, Qian J, Fang DY, Yu Y, Zhi JF 2016 Development of a mediated whole cell-based electrochemical biosensor for joint toxicity assessment of multi-pollutants using a mixed microbial consortium. Anal. Chim. Acta 924, 21–28. (10.1016/j.aca.2016.04.011) 27181640

[30] Cha KH, Jensen GC, Balijepalli AS, Cohan BE, Meyerhoff ME 2014 Evaluation of commercial glucometer test strips for potential measurement of glucose in tears. Anal. Chem. 86, 1902–1908. 24428813 10.1021/ac4040168

[31] Wang XJ, Liu M, Wang X, Wu Z, Yang LZ, Xia SQ, Chen L, Zhao JF 2013 *P*-benzoquinone-mediated amperometric biosensor developed with *Psychrobacter,* sp. for toxicity testing of heavy metals. Biosens. Bioelectron. 41, 557–562. (10.1016/j.bios.2012.09.020) 23062555

[32] Dalzell DJB et al. 2002 A comparison of five rapid direct toxicity assessment methods to determine toxicity of pollutants to activated sludge. Chemosphere 47, 535–545. (10.1016/S0045-6535(01)00331-9) 11996129

[33] Li JM, Yu Y, Qian J, Wang Y, Zhang JH, Zhi JF 2014 A novel integrated biosensor based on co-immobilizing the mediator and microorganism for water biotoxicity assay. Analyst 139, 2806–2812. (10.1039/c4an00243a) 24728093

[34] Fang DY, Gao GY, Shen J, Yu Y, Zhi JF 2016 A reagentless electrochemical biosensor based on thionine wrapped *E. coli*, and chitosan-entrapped carbon nanodots film modified glassy carbon electrode for wastewater toxicity assessment. Electrochim. Acta 222, 303–311. (10.1016/j.electacta.2016.10.174)

[35] Gao GY, Fang DY, Yu Y, Wu LZ, Wang Y, Zhi JF 2017 A double-mediator based whole cell electrochemical biosensor for acute biotoxicity assessment of wastewater. Talanta 167, 208–216. (10.1016/j.talanta.2017.01.081) 28340712

[36] Zeb BS, Ping Z, Mahmood Q, Lin Q, Pervez A, Irshad M, Bilal M, Bhatti ZA, Shaheen S 2016 Assessment of combined toxicity of heavy metals from industrial wastewaters on *Photobacterium phosphoreum*, T_3_S. Appl. Water Sci. 7, 1–8. (10.1007/s13201-016-0385-4)

[37] Hassan SH, Oh SE 2010 Improved detection of toxic chemicals by *Photobacterium phosphoreum* using modified Boss medium. J. Photochem. Photobiol. B Biol. 101, 16–21. (10.1016/j.jphotobiol.2010.06.006) 20637650

[38] Chen C, Xie Q, Wang L, Qin C, Xie F, Yao S, Chen J 2011 Experimental platform to study heavy metal ion-enzyme interactions and amperometric inhibitive assay of Ag**^+^** based on solution state and immobilized glucose oxidase. Anal. Chem. 83, 2660–2666. (10.1021/ac1031435) 21391572

[39] Chen Y, Wang Z, Chu J, Zhuang Y, Zhang S, Yu X 2013 Significant decrease of broth viscosity and glucose consumption in erythromycin fermentation by dynamic regulation of ammonium sulfate and phosphate. Bioresour. Technol. 134, 173–179. (10.1016/j.biortech.2013.02.023) 23500575

[40] Fang DY, Yu Y, Wu LZ, Wang Y, Zhang JH, Zhi JF 2015 Bacillus subtilis-based colorimetric bioassay for acute biotoxicity assessment of heavy metal ions. RSC Adv. 5, 59 472–59 479. (10.1039/c5ra05452d)

[41] Wang ZZ, Chen ZW, Gao N, Ren JS, Qu XG 2015 Transmutation of personal glucose meters into portable and highly sensitive microbial pathogen detection platform. Small 11, 4970–4975. (10.1002/smLl.201500944) 26153225

[42] Yip NC, Rawson FJ, Tsang CW, Mendes PM 2014 Real-time electrocatalytic sensing of cellular respiration. Biosens. Bioelectron. 57, 303–309. (10.1016/j.bios.2014.01.059) 24607581 PMC3990025

[43] Novelli AA, Losso C, Ghetti PF, Ghirardini AV 2010 Toxicity of heavy metals using sperm cell and embryo toxicity bioassays with *Paracentrotus lividus* (Echinodermata: Echinoidea): comparisons with exposure concentrations in the Lagoon of Venice, Italy. Environ. Toxicol. Chem. 22, 1295–1301. (10.1002/etc.5620220616) 12785587

[44] Catterall KP, Robertson D, Hudson S, Teasdale PR, Welsh DT, John R 2010 A sensitive, rapid ferricyanide-mediated toxicity bioassay developed using *Escherichia coli*. Talanta 82, 751–757. (10.1016/j.talanta.2010.05.046) 20602965

[45] Shen GQ, Lu YT, Zhou QX, Hong JB 2005 Interaction of polycyclic aromatic hydrocarbons and heavy metals on soil enzyme. Chemosphere 61, 1175–1182. (10.1016/j.chemosphere.2005.02.074) 16263387

[46] Krznaric E, Wevers JH, Cloquet C, Vangronsveld J, Vanhaecke F, Colpaert JV 2010 Zn pollution counteracts Cd toxicity in metal-tolerant ectomycorrhizal fungi and their host plant, *Pinus sylvestris*. Environ. Microbiol. 12, 2133–2141. (10.1111/j.1462-2920.2009.02082.x) 21966908

[47] Wang YX, Chen ML 2011 Variation of the fraction transformations and mobility of Cu and Zn in municipal sludge with pH. In International Conference on Mechanic Automation & Control Engineering, pp. 3741–3744. (10.1109/MACE.2011.5987809)

