# Supplementary material for: Acute biotoxicity assessment of heavy metals in sewage sludge based on the glucose consumption of Escherichia coli
Source: R Soc Open Sci. 2019 Jan 23;6(1):181769. doi: 10.1098/rsos.181769 (PMC6366162; doi:10.1098/rsos.181769)
Supplement: The parameter optimization of heavy metal extract solution in spiked sludge based on the glucose consumption of Escherichia coli [file rsos181769supp1.doc]

**Supplement Material:**

In the experiment of biotoxicity assessment of heavy metals in sludge, the parameter optimization process of the heavy metal extract solution was instructed in figure S1. Selected industrial park sludge with Zn contamination to prepared spiked sludge, in the process, the mixed ratio of real sludge and fresh soil were prepared as 1: 4, 1: 9 and 1: 19, respectively. Hydrochloric acid (0.1 mol L^-1^), acetic acid (0.1 mol L^-1^) and deionized water have been selected as the heavy metal extract agents. Took dry spiked sludge samples and heavy metal extract agent into centrifuge tube, then centrifuged (180 r min^-1^) it for 12 h to obtain the heavy metal extract solution. Took out 5 μL supernatants from the heavy metal extract solution and measured the glucose concentration by PGM. The glucose metabolism inhibition of *E. coli* in spiked sludge with Zn contamination by hydrochloric acid was 38.4%, which was higher than acetic acid and deionized water was shown in figure S1(a). In figure S1(b), with the decreased of the mixed ratio of industrial park sludge and fresh soil, the glucose metabolism inhibition of *E. coli* was increased firstly and then decreased. The highest glucose metabolism inhibition of *E. coli* was 54%, which had appeared in 1:9 mixed ratio. Equilibrium the heavy metal extract solution for 12 h, the system was balanced from 2 h as shown in figure S1(c). According to the parameter optimization process of heavy metal extract solution in sludge, Zn ion extract solution has the highest biological inhibition to glucose metabolism of *E. coli*.


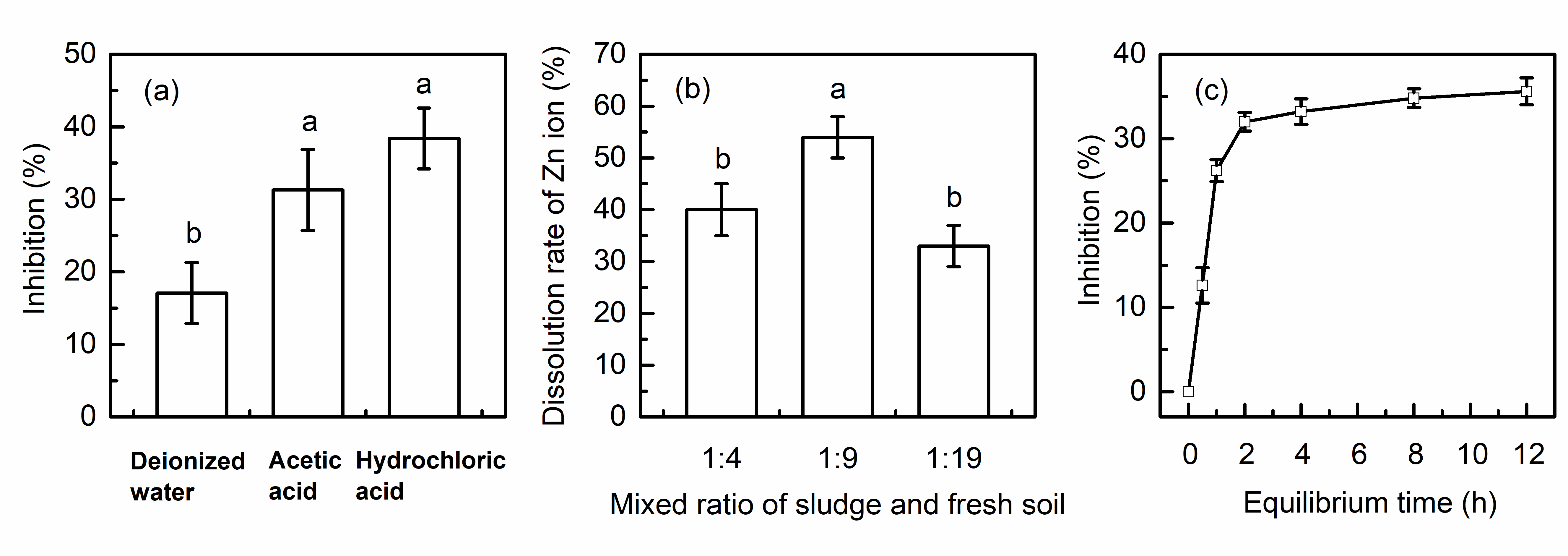


Figure S1. The parameter optimization of heavy metal extract solution in sludge with Zn contamination: (a) The glucose metabolism inhibition of *E. coli* with different extract agents; (b) The dissolution rate of Zn ion with different mixed ratio of real sludge and fresh soil; (c) The glucose metabolism inhibition of *E. coli* during all the equilibrium time. Data points represent the average of three replicates.
